# Astrocyte and microglial activation in the lateral geniculate nucleus and visual cortex of glaucomatous and optic nerve transected primates

**Published:** 2009-10-31

**Authors:** Dawn Lam, Janey Jim, Eleanor To, Carol Rasmussen, Paul L. Kaufman, Joanne Matsubara

**Affiliations:** 1 Southern California College of Optometry, Fullerton, CA; 2 Department of Ophthalmology and Visual Sciences, University of British Columbia, Vancouver, British Columbia, Canada; 3 Department of Ophthalmology and Visual Sciences, University of Wisconsin-Madison, Madison, WI

## Abstract

**Purpose:**

To examine early cellular changes, including astrocyte reactivity and microglial activation, in the central nervous system (CNS) after unilateral optic nerve transection (ONT) or ocular hypertension (OHT) in monkeys.

**Methods:**

Unilateral ONT or OHT was achieved in monkeys for periods ranging from two weeks to two months in duration. After intracardial perfusion, sections of the lateral geniculate nucleus (LGN) and visual cortex (V1) were examined by immunohistochemistry for glial fibrillary acidic protein (GFAP) and CD11b, a subunit of the complement 3 receptor and marker of macrophage and microglia cells (MAC-1). Alternate serial sections were evaluated by cytochrome oxidase (CO) histochemistry to assess metabolic activity.

**Results:**

Both ONT and OHT caused a reduction in metabolic activity in the treated eye layers of the LGN and V1. GFAP and MAC-1 immunoreactivities were elevated in spatial register with the treated eye layers of the LGN and V1 in ONT animals. In the OHT animals, GFAP, but not MAC-1, immunoreactivity was elevated in spatial register with the treated eye layers of LGN and V1. Thus, during the first weeks after OHT or ONT, loss of metabolic activity was accompanied by astrocyte and microglial activation in the ONT group and astrocyte activation in the OHT animals.

**Conclusions:**

These results suggest that unilateral OHT or ONT triggers separate signaling pathways that promote differential activation of CNS glial populations. Astrocyte reactivity was present in all brains studied and demonstrates the loss of metabolic activity is accompanied by increased GFAP immunoreactivity. Microglial activation was only observed in ONT brains. The lack of microglial activation as late as two months following OHT may represent a time window for early treatment to prevent long-term neuronal loss in the CNS after OHT.

## Introduction

Glaucoma is a condition which causes a loss of retinal ganglion cell activity. This loss has been shown to cause neurochemical changes in the lateral geniculate nucleus (LGN) and visual cortex of adult primates. Studies have revealed that the activity of the mitochondrial enzyme, cytochrome oxidase (CO) is reduced after monocular visual deprivation by lid suture (MD), tetrodotoxin (TTX) injection, enucleation, and optic nerve transection (ONT) in adult cats and primates [1-8]. The development of an experimental ocular hypertension (OHT) model using primates, in which intraocular pressure (IOP) is elevated, allows us to study the effects of a visual loss that more closely mimics the neurodegenerative processes associated with human glaucoma [9,10]. Using such primate models, it was shown that elevation of IOP causes selective damage of retinal ganglion cells [11-14], and alterations in the neuronal structure and neuromodulatory chemicals in the LGN [15-20] and in V1 of the central nervous system (CNS) [21-23]. In glaucoma patients, elevated IOP may cause axonal dysfunction with or without retinal ganglion cell loss, leading to subsequent visual field defects [24,25] and central changes in the LGN and V1 [26-29].

Several studies reported that elevated IOP lasting for one year or more results in neurodegenerative events including neuronal atrophy, shrinkage of dendritic arbors, and a reduction in both the number and diameter of neurons in the retina and brain [12-14,18-20]. These studies highlight the later stages of neurodegeneration in the CNS after vision loss by long-term elevated IOP. As the loss of neurons is irreversible in the adult CNS, there is an important need to focus on the early events that take place in the CNS after elevated IOP before cell loss, to develop strategies to prevent neuronal atrophy and loss both in the eye and the brain.

Among the earliest cellular events after sensory deafferentation are the loss of CO activity and the activation of glial cells, which have been shown to occur within hours after action potential blockage by TTX injection or deafferentation [3,30-32]. In the CNS, glial cells outnumber neurons by a factor of ten and serve a supportive role by surrounding neuronal cell bodies, axons, and dendrites. Their function ranges from providing structural support in the normal healthy CNS, to removing debris after cell death and injury. A major class of glial cells, the astrocyte, functions in daily maintenance of the extracellular milieu and also regulates the cerebral vasculature and neuronal activity [33-36]. In response to traumatic injury, astrocytes multiply, increase the production of intermediate filaments, and later form dense gliotic scars to contain damaged tissues [37-41]. Another major class of glial cell, the microglia, monitors ion levels and neurotransmitter changes in the extracellular matrix [42-47]. In their activated state, microglia proliferate and migrate to sites of injury or disease, where they remove cellular debris [46,48]. Activated microglia also produce inflammatory cytokines that promote microglial and macrophage migration, and may ultimately result in secondary neuronal damage due to the generation of reactive oxygen species via respiratory burst mechanisms and thus, may themselves promote inflammation and neurodegeneration.

Little is known of the glial response in the CNS of primates after elevated IOP; however, earlier studies in rodents revealed that microglial cells become activated and lead to neuronal cell death in the LGN [49]. The objective of this study was to identify the early changes in neuronal activity and glial activation in the LGN and V1 after short-term unilateral elevated IOP (two weeks to two months) in primates with OHT. For comparison, we also studied the glial response to ONT, a form of deafferentation that is known to cause immediate and irreversible, transsynaptic (anterograde) degeneration in the CNS. Understanding the neurochemical changes in the CNS after short-term elevated IOP may lead to developing strategies for early detection or protection of surviving neurons, both in the retina and brain, from neurodegeneration associated with glaucoma.

## Methods

This study used eight rhesus (*Macaca mulatta*) and four cynomolgus (*Macaca fascicularis*) monkeys that were ages 3 to 24 years. All 12 male and female monkeys were treated at the University of Wisconsin, Madison, WI. Animals were acquired from Covance Labs, Madison, WI, Wisconsin National Primate Research Center, Madison, WI or Bioqual, Rockville, MD. All monkeys were housed in standard 12 h cycle lighting with water ad libitum and food provided twice a day. The enrichment program included social housing, toys/objects to manipulate, cage furniture, foraging devices, fruit, and other nutritive snacks, radio and TV/movies. All experiments were done in accordance with the ARVO Statement for the Use of Animals in Ophthalmic and Vision Research as well as with National Institutes of Health and University of Wisconsin guidelines.

### Animal surgical procedures

Two rhesus and two cynomolgus monkeys underwent laser ablation of the trabecular meshwork [10,50] in one eye, which resulted in ipsilateral elevated IOP for periods of two to eight weeks (Table 1). A standard clinical argon laser and slit lamp delivery system was used to produce a series of 75 to 250 focal lesions to the trabecular meshwork. The laser was set to deliver a 50 μm spot diameter at 1–1.5 W with a 0.5 s duration. Two additional animals had spontaneous unilateral elevation of IOP. These animals had developed unilateral secondary glaucoma due to persistent inflammation following intracameral injection of different experimental drugs. IOP was monitored every three to ten days under intramuscular (IM) injection of 10 mg/kg ketamine anesthesia (3–5 mg/kg supplement as needed) using a minified Goldmann applanation tonometer [51] (Haag-Streit, Koniz, Switzerland). These measurements were occasionally backed up by measurements with a handheld applanation tonometer (Tono-pen XL; Mentor O & O, Norwell, MA). Tonopen measurements were converted to actual mmHg based on a standard calibration curve [52]. IOP was measured with the monkey lying prone in a head holder. If IOP was not consistently above 30–35 mmHg, additional laser treatments were performed until stable ocular hypertension was achieved. IOP was checked every three to ten days thereafter to assure stability. Additional laser treatment or IOP lowering therapy was applied as needed to maintain IOP at the desired level. If the IOP was higher than the protocol target range or if there was any sign of discomfort due to elevated IOP, the monkeys were treated topically once or twice daily with a single drop of one or more of the following until the desired IOP was achieved: 0.5% timolol maleate in gel-forming vehicle (Timoptic-XE; Merck & Co., Whitehouse Station, NJ), 0.2% brimonidine tartrate (Alphagan; Allergan, Irvine, CA), 2% dorzolamide hydrochloride (Trusopt; Merck & Co.), and 2 μg prostaglandin F2 alpha-isopropyl ester (donated by Pharmacia Corp, Peapack, NJ). If necessary, 5 mg/kg IM acetazolamide sodium (Ben Venue Laboratories, Bedford, OH) was given once or twice daily. The opposite eye served as a normal control eye.

**Table 1:** Animal table: optic nerve transection and ocular hypertension.

**Optic Nerve Transection**											
**ID#**	**Species**	**Duration of treatment**	**Treated eye**	**Hemisphere processed**	** **	** **	** **	**Immunoreactivity in treated eye band**			
** **	** **	** **	** **	** **	** **	** **	** **	**GFAP**		**Mac-1**	
** **	** **	** **	** **	** **	** **	** **	** **	**LGN**	**V1**	**LGN**	**V1**
ONT 1 (AP75)	Rhesus	14 days	R	R	** **	** **	** **	NS	++	NS	NS
ONT 2 (32676)	Rhesus	14 days	R	L	** **	** **	** **	+	++	+	++
ONT 3 (AR 96)	Rhesus	28 days	R	L	** **	** **	** **	++	++	NS	NS
ONT 4 (19069)	Rhesus	28 days	R	L	** **	** **	** **	++	NS	NS	NS
ONT 5 (AP39)	Rhesus	28 days	L	R	** **	** **	** **	NS	+	NS	++
ONT 6 (Rh168)	Rhesus	28 days	R	L	** **	** **	** **	++	++	+	+
**Ocular Hypertension (>30 mmHg)**											
ID#	Species	**Duration**	**Tx eye mean IOP mmHg (C eye)**	**Tx eye cup/disk (C eye)**	**Integral**	**Treated eye**	**Hemisphere processed**	**Immunoreactivity in treated eye band**			
** **	** **	** **	** **	** **	** **	** **	** **	**GFAP**		**Mac-1**	
** **	** **	** **	** **	** **	** **	** **	** **	**LGN**	**V1**	**LGN**	**V1**
GL 1 (534)	Cynomolgus	14 days	48 (16)	0.4 (0.4)	672	R	L	NS	+	NS	NS
GL 2 (452)	Cynomolgus	40 days	51 (16)	na (0.3)	2040	R	L	NS	++	NS	NS
GL 3 (AI 34)	Rhesus	55 days	46 (18)	0.9 (0.2)	2530	R	R	++	++	-	-
GL 4 (529)	Cynomolgus	56 days	47 (14)	0.9 (0.2)	2632	R	R	++	++	-	-
GL 5 (570)	Cynomolgus	45 days	40 (17)	0.9 (0.3)	1800	R	R	NS	++	NS	NS
GL 6 (AS02)	Rhesus	58 days	35 (18)	0.5 (0.3)	2030	L	L	++	++	-	-

This table identifies treatment, duration of treatment, and results from analysis of tissue processed for GFAP and Mac-1 immunoreactivity. Abbreviations: tissue not stained (NS), robust immunostaining (++), moderate immunostaining (+).

Quantification of vision loss experienced by animals with unilateral ocular hypertension was calculated using an “integral” value, defined as the number of days of IOP measurements >30 mmHg multiplied by the mean IOP value for that period of time [53]. Vision loss by ONT was quantified by the total number of days after ONT surgery and before euthanasia. A summary of these values is shown in Table 1.

Six additional rhesus monkeys underwent unilateral optic nerve transection [54] (Table 1). Animals were given an initial IM injection of 10–15 mg/kg ketamine, which was followed by intubation, then general anesthesia under 1%–3% isoflurane gas. After surgery, monkeys were treated with systemic benzathine and 30,000 U/kg procaine penicillin for five days and 1mg/kg IM methylprednisolone acetate for three weeks, tapering to 0.1 mg/kg for 1 more week. As an analgesia, 0.1 mg/kg IM injection of buprenorphine was given for three days. Two animals were euthanized with 1 ml/4.5 kg intravenous Euthasol (each milliliter contains 390 mg of pentobarbital and 50 mg of phenytoin) at two weeks following the surgery, and the remaining four animals were euthanized at four weeks following ONT surgery.

### Tissue processing

All twelve animals were euthanized and perfused intracardially with 750 ml phosphate buffer saline (PBS; 9 g/l NaCl, 3.96 g/l NaH_2_PO_4_-H_2_O, and 22.68 g/l HNa_2_O_4_P-7H_2_O), followed by one liter of 4% paraformaldehyde and again with 200–300 ml PBS following 25 mg/kg IV pentobarbital anesthesia. The brain was removed and placed in a solution of 10% sucrose in phosphate buffer and shipped on ice packs by overnight courier to the University of British Columbia. Upon arrival, area V1 and LGN sections were dissected from the rest of the brain. Area V1 was blocked such that it could be gently flattened tangentially between two glass slides before being frozen on dry ice and stored at −80 °C. The LGN was blocked in the coronal plane, frozen on dry ice and stored at −80 °C as previously described [23].

### CO histochemistry and immunohistochemistry

Tissue blocks were cut tangentially at 50 μm on a freezing microtome. Alternate 50 μm sections were stained for CO activity using previously-published protocols [23,55]. Briefly, 20 mg of diaminobenzidine (DAB; Sigma-Aldrich, St. Louis, MO) was dissolved in 50 ml of distilled water. Once dissolved, 50 ml (0.1 M, pH 7.2) phosphate buffer (PB), 2 g sucrose, 30 mg cytochrome C (Sigma-Aldrich) derived from horse heart and 20 mg of catalase (Sigma-Aldrich) derived from bovine liver were added to the DAB solution. Then, 5 ml of 1% nickel ammonium sulfate was added dropwise followed by approximately 3 ml of 1% cobalt chloride until the solution appeared slightly opaque. Tissue sections were placed into 12-well plates filled with 1.5 ml of the cytochrome oxidase solution and incubated at 37 °C for 30–45 min. Upon completion of the reaction, sections were washed three times for 5 min each in PB. Then the sections were mounted onto glass slides and air dried overnight. Mounted sections were then dehydrated in a series of graded alcohols and cleared in xylene. Mounting medium, Permount (Fisher Scientific, Waltham MA) was applied onto sections and then coverslipped.

Monoclonal antibodies against human CD11b (MAC-1; Cederlane Labs, Burlington, VA) and glial fibrillary acidic protein (GFAP; Chemicon, Temecula, CA) were used to identify activated microglial and astrocytes, respectively. Free-floating sections were incubated in 3% normal horse serum (NHS) for 1 h to block nonspecific binding, and then washed three times for 5 min each in PB before incubating in primary antibodies. Primary antibodies against MAC-1 and GFAP were used at a 1:300 dilution in 3% NHS in 0.3% Triton X-100 (TX-100) made in PB. After rinsing, sections were incubated at 4 °C in primary antibody for 36 to 48 h with agitation. They were then rinsed three times for 5 min each in PB before incubating in 0.1% secondary antibody (biotinylated anti-mouse made in horse) in 3% NHS and 0.3% TX-100 in PB for 2 h at room temperature. Next, sections were placed in a solution of avidin biotin complex (Vector Laboratories, Burlingame, CA) for 1 h. Visualization of the antibody binding was undertaken using a glucose oxidase driven DAB as described previously [23,55]. Sections were mounted on gelatin-coated slides, air-dried, dehydrated, cleared in xylene and coverslipped with Permount (Fisher Scientific). Negative control sections were processed after the primary antibody was replaced with a solution containing 3% NHS and 0.3% TX-100.

### Data collection and analysis

**Figure 1:**
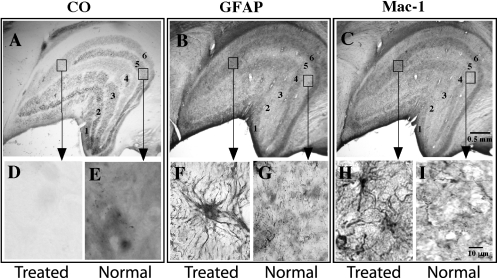
Photomicrographs of serial coronal sections of LGN from an ONT brain. **A**: Section stained for CO histochemistry demonstrates reduced CO activity in contralateral (Layers 1,4,6) eye layers. **B**: Serial section stained for GFAP immunohistochemistry demonstrates denser reaction product in the contralateral eye layers. **C**: Serial sections stained for MAC-1 immunohistochemistry demonstrates a denser staining pattern in the contralateral eye layers. Arrows point to high power photographs (100X) of treated versus normal LGN layers stained for CO (**D**, **E**), GFAP (**F**, **G**), or MAC-1 (**H**, **I**). Note astrocytic profiles and processes in **F** and microglial profiles in **H**.

**Figure 2:**
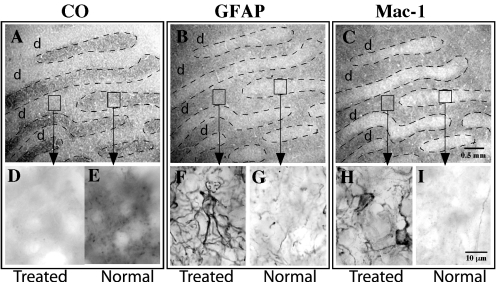
Photomicrographs of serial tangential sections through layer 4C of V1 from an ONT brain. **A**: Section stained for CO histochemistry demonstrates ocular dominance bands in tangential sections through layer 4c of V1. Note the treated eye bands (d) demonstrate lighter, less dense CO staining. **B**: A serial section stained for GFAP immunohistochemistry demonstrates denser, more robust immunoreactivity in the ocular dominance bands associated with the treated eye (d). **C**: A serial section stained for MAC-1 immunohistochemistry demonstrates more robust immunoreactivity in the ocular dominance bands associated with the treated eye (d). Arrows point to high power photographs (100X) are shown and contrast staining in treated and normal eye bands for CO (**D, E**), GFAP (**F, G**), or MAC-1 (**H, I**) immunoreactivity. Note astrocytic profiles and processes in **F** and microglial profiles in **H**.

**Figure 3:**
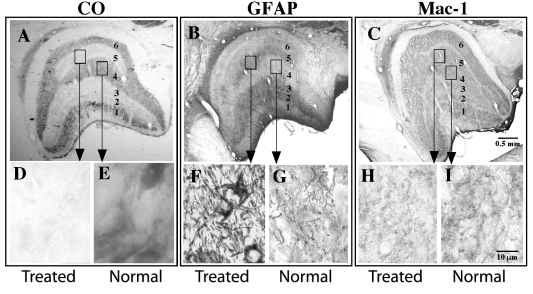
Photomicrographs of coronal sections of LGN from OHT brains. **A**: Section stained for CO histochemistry demonstrates reduced CO activity in ipsilateral eye layers (Layers 2, 3, 5). **B**: Serial section stained for GFAP immunohistochemistry demonstrates denser reaction product in the contralateral eye layers (Layers 1, 4, 6). **C**: Section stained for MAC-1 immunohistochemistry demonstrating uniform labeling throughout all LGN layers. Arrows point to high power photographs (100X) are shown and contrast staining in treated and normal eye bands for CO (**D**, **E**), GFAP (**F**, **G**), or MAC-1 (**H**, **I**) immunoreactivity. Note astrocytic profiles and processes in **F** and lack of labeled profiles in **H** and **I**.

**Figure 4:**
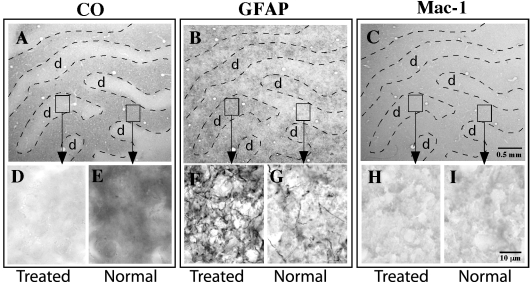
Photomicrographs of serial tangential sections through layer 4C of V1 from an OHT brain. **A**: Section stained for CO histochemistry demonstrates ocular dominance bands in tangential sections through layer 4C of V1. Note the treated eye bands (d) demonstrate lighter, less dense CO staining. **B**: A serial section stained for GFAP immunohistochemistry demonstrates denser, more robust immunoreactivity in the ocular dominance bands associated with the treated eye (d). **C**: A serial section stained for MAC-1 immunohistochemistry demonstrates uniform immunoreactivity. Arrows point to high power photographs (100X) of the treated and normal ocular dominance bands stained for CO (**D**, **E**), GFAP (**F**, **G**), or MAC-1 (**H**, **I**). Note astrocytic profiles and processes in **F** and lack of labeled microglial profiles in **H** and **I**.

**Figure 5:**
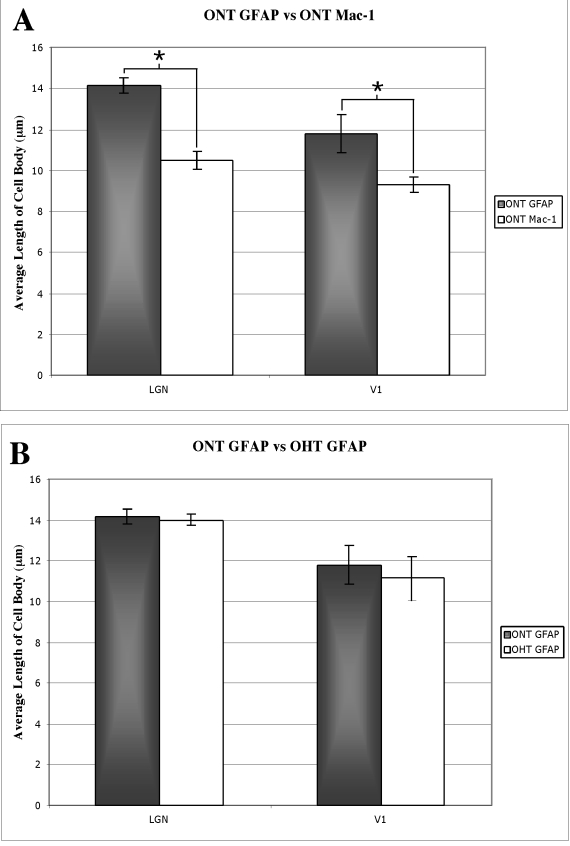
GFAP and MAC-1 staining in the LGN and area V1. **A**: In the LGN of the ONT brains, GFAP immunoreactive cells (black bars, 14.2±0.4 SD) are larger than the MAC-1 immunoreactive cells (white bars, 10.5±0.4 SD). In V1, GFAP immunoreactive cells were again larger (black bar, 11.8±0.9 SD) than the MAC-1 immunoreactive cells (white bar, 9.5±0.4 SD). Asterisks indicate significance at the p<0.05 level. **B**: A comparison of the GFAP immunoreactive cells in the LGN of ONT (14.2±0.4 SD) and OHT (14.0±0.3 SD) brains revealed that the two populations were indistinguishable based on cell size. A similar comparison of GFAP immunoreactive cells in V1 of ONT (11.8±0.9 SD) and OHT (11.1±1.1 SD) revealed no difference in the size range in GFAP immunoreactive cells in V1.

Images of processed LGN and V1 sections were captured using standard digital camera settings and 1×, 20×, 40×, and 100× objective lenses attached to an upright, bright-field compound microscope (Nikon, Tokyo Japan). Serial sections were aligned using the pattern of blood vessels as fiduciary landmarks. High power images of GFAP and MAC-1 immunoreactivity (Figure 1D-I, Figure 2D-I, Figure 3D-I, and Figure 4D-I) were taken using a 100× oil objective lens. A semiquantitative analysis of the immunoreactivity was assessed using three microscope fields in each of three tissue sections for a total of nine microscope fields. The intensity of immunoreactivity in the treated eye layers was scored in a semiquantitative manner. Background immunoreactivity was represented with a minus sign (−). One plus (+) represented moderate immunoreactivity, while two pluses (++) represented significant immunoreactivity (see Table 1). The diameters of the immunoreactive cell profiles were measured using a 40× objective and 10× eyepieces and expressed as mean±SD (Figure 5). A paired *t*-test (Minitab Statistical Software, State College, PA) was used to compare the measurements of cell profile diameters for GFAP and MAC −1 populations and a two sample *t*-test (Minitab Statistical Software) was used to compare the GFAP populations between ONT and OHT tissues.

## Results

### Animals with optic nerve transection

CO histochemistry revealed light CO staining in layers 1, 4, and 6 of the LGN, a pattern that confirmed that the transected optic nerve (right) was contralateral to the processed hemisphere (left) in five of the six brains (Figure 1A,D,E). CO staining in layers 2, 3, and 5, the ipsilateral layers of the LGN, demonstrated darker CO staining and represented normal levels of CO activity in both the 14 day and the 28 day post ONT brains. In one animal (AP75), the hemisphere ipsilateral to the transected nerve was processed; CO histochemistry revealed light CO staining in the ipsilateral layers of the LGN (layers 2, 3, and 5). The relative density of the CO staining in the treated magnocellular and parvocellular layers of the LGN was equally reduced after ONT. CO histochemistry of tangential sections through V1 revealed a series of lightly and darkly staining ocular dominance bands in layer 4C (Figure 2A,D,E) and blobs in layer 2/3 (not shown), reflecting the transsynaptic loss of geniculocortical afferent activity associated with the transected nerve. This pattern of CO staining in the LGN and V1 is consistent with earlier studies [1,4,5,7,8] in which animals were subjected to ONT or MD by lid suture.

**Figure 6:**
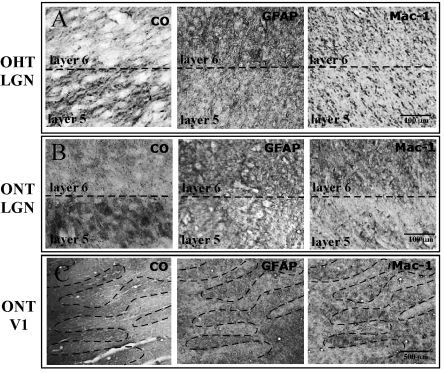
CO histochemistry, GFAP and MAC-1 immunohistochemistry in unilateral ONT and OHT brains. After 14 days, the CO staining demonstrated lighter staining in the contralateral (treated) eye bands (Layer 1, 4, 6) in the LGN of both the OHT (534) and the ONT (32676) brains, consistent with the findings from animals with vision loss of longer duration (Figures 1–4). The GFAP immunoreactivity was stronger in the regions of LGN and V1 associated with the treated eye in both OHT (**A**) and ONT (**B**, **C**) brains. The MAC-1 immunoreactivity was stronger in the regions associated with the treated eye in ONT (**B**, **C**) brains only. MAC-1 immunoreactivity did not show differential expression between treated and normal ocular dominance bands, consistent with findings from long-term OHT (Figure 3 and Figure 4).

Astrocytic reactivity was assessed with GFAP immunoreactivity. All ONT brains processed for GFAP immunoreactivity demonstrated a pattern of robust labeling in the treated eye layers of the LGN (n=4) and the treated ocular dominance bands of area V1 (n=5; Figure 1B,F,G; Figure 2B,F,G, and Figure 6B,C). GFAP immunoreactivity revealed cytoplasmic labeling of astrocyte somata and their highly ramified processes (Figure 1F and Figure 2F). The mean diameter of the GFAP immunoreactive cell bodies in the parvocellular layers of the LGN was 14.2 µm (±0.4 SD). The mean diameter of the GFAP immunoreactive profiles in V1 was 11.8 µm (±0.9 SD). GFAP immunoreactivity also resulted in strong labeling of highly ramified processes that surrounded what were presumed to be unlabeled neuronal profiles.

LGN layers receiving input from the intact optic nerve and the ocular dominance bands in V1 associated with the normal eye were significantly less immunoreactive for GFAP. At high power, GFAP immunoreactivity in the normal eye layers/bands demonstrated sparse labeling of thin, astrocytic processes and few somatic profiles (Figure 1G and Figure 2G).

Microglial activation was assessed by immunoreactivity to MAC-1. MAC-1, also known as CD 11b, is a component of the complement receptor C3b. All ONT brains processed for MAC-1 immunoreactivity demonstrated robust labeling in the treated layers/bands in the LGN (n=2) and V1 (n=3; Figure 1C,H, Figure 2C,H, and Figure 5B,C). Both the cytoplasm and processes of microglial cells displayed robust immunoreactivity. The diameter of the immunoreactive profiles in the LGN and V1 were 10.5 µm (±0.4 SD) and 9.5 µm (±0.4 SD), respectively. In the normal eye layers/bands of the LGN and V1, few immunoreactive cell bodies or processes were present (Figure 1I and Figure 2I,).

We performed a paired *t*-test to verify that, based on cell diameter, the population of GFAP cells was distinct from the population of MAC-1 immunoreactive cells in the LGN of ONT brains (p<0.05). This test also confirmed that GFAP and MAC-1 positive cells were from separate populations in V1 of ONT brains (p<0.05; Figure 6A).

### Animals with unilateral ocular hypertension

Vision loss by unilateral OHT caused a downregulation of CO activity in the LGN layers innervated by the OHT eye in all six brains processed. Each animal experienced different degrees of IOP elevation, ranging from 35 to 51 mmHg. Furthermore, the durations of elevated IOP also differed among the animals, ranging from 14 to 58 days. The calculated integral values, which reflect the relative severity of OHT, ranged from 672 to 2,632 (Table 1). The fluctuations in CO density between the treated and normal eye layers of the LGN were of similar relative contrast among all six brains. The example shown in Figure 3A is from an animal in whom the ipsilateral hemisphere was processed, and thus the downregulation in CO activity was evident in layers 2 (magnocellular) and layers 3, 5 (parvocellular) of the LGN (Figure 3D,E). The example shown in Figure 5A is from an animal in whom the contralateral hemisphere was processed, and thus the downregulation in CO activity was evident in layers 1, 4, and 6. CO histochemistry of tangential sections through V1 revealed a series of lightly and darkly stained ocular dominance bands in layer 4C (Figure 4A), reflecting a transsynaptic reduction in activity of the geniculocortical afferent pathway associated with the OHT eye. The observed pattern of CO staining in the LGN and V1 was consistent with the results after vision loss by ONT as described above and consistent with earlier studies after short-term and long-term unilateral OHT [21-23].

Astrocyte activation after unilateral OHT was assessed with GFAP immunoreactivity. All OHT brains processed for GFAP immunoreactivity demonstrated robust immunostaining in the treated eye layers of the LGN (n=3), and moderate to robust immunostaining in the treated ocular dominance bands of V1 (n=5; Figure 3B, Figure 4B, and Figure 5A). Higher power images of GFAP immunohistochemistry demonstrated labeling of astrocytic processes often surrounding what were presumed to be unlabeled neuronal profiles (Figure 3F and Figure 4F). The mean diameter of the GFAP immunoreactive cell bodies in the parvocellular layers of the LGN was 14.0 µm (±0.3 SD), while the mean diameter of the GFAP immunoreactive profiles in V1 was 11.1 µm (±1.1 SD). The normal eye layers/bands in the LGN and V1 displayed background levels of immunoreactivity for GFAP; this was evident particularly at low power (Figure 3B, Figure 4B, and Figure 5A). At high power, GFAP immunoreactivity in the normal eye layers and ocular dominance bands displayed occasional thin, astrocytic processes that were labeled, but these processes rarely outlined presumably unlabeled neuronal profiles (Figure 3G and Figure 4G). We performed a two-sample *t*-test to determine, based on cell diameter, whether the GFAP immunoreactive cells in the OHT and the ONT brains were from the same population. The tests demonstrated that, based on cell diameter, GFAP immunoreactive cells in the LGN of the ONT and OHT brains were from the same population (p=0.595). This was also confirmed for the GFAP immunoreactive cells in V1 of the ONT and OHT (p=0.476; Figure 6B).

MAC-1 immunoreactivity was at background levels in the LGN (Figure 3C and Figure 5A) and V1 (Figure 4C) of the animals with unilateral OHT. There were no fluctuations in immunoreactivity between treated and normal eye layers of the LGN or in the ocular dominance bands in V1; there was a uniform staining throughout the LGN and V1 of all animals tested (n=3; Figure 3H,I and Figure 4H,I).

## Discussion

Glaucomatous loss of retinal ganglion cells can cause neurodegeneration in the central visual pathways in animal models [15-20] and in humans [26,27,56]. An understanding of the early events associated with neurodegeneration that may take place in the CNS after elevated IOP would be useful in developing strategies to prevent atrophy of central neurons in the LGN and V1. Our study is significant in that it is one of the first to examine early metabolic activity loss and glial response in the CNS within the initial 60 days after unilateral elevated IOP in a primate model of OHT. Effects of elevated IOP on retinal ganglion cells in this primate model are usually slow and progressive, and thus mimic the human disease better than other, more severe models that result in retinal ganglion cell death over shorter time frames. For comparison, we also studied the metabolic activity and the glial responses after unilateral ONT. ONT results in neuronal cell loss by transsynaptic (anterograde) degeneration in the CNS.

Our findings revealed a substantial drop in metabolic activity, as assessed with CO histochemistry, in the treated eye bands of the LGN and V1 at all time points studied for both OHT and ONT. Loss of CO activity in the CNS after monocular vision loss in the adult primate has been documented as early as 14 h after an intraocular TTX injection, and thus represents one of the earliest measurable responses to loss of visual inputs to the retinogeniculocortical pathway [57]. Measurement of decreased neuronal metabolic activity may therefore have value as a surrogate for determining functional loss in the CNS [29].

In this study, we were able to assess a concomitant rise in GFAP immunoreactivity, a marker of astrocyte activation, in spatial register with treated eye bands in both ONT and OHT animals. Astrocytes are known to influence synaptic activity by forming glutamate precursors [58,59]. Gordon et al. found that astrocytes can also modulate neuronal activity indirectly by changing the blood flow and the cerebral vasculature [33]. Astrocytic reactivity after sensory deafferentation was shown in the chick cochlear nucleus and in the rat LGN after blockade of action potentials by TTX injections and after enucleation [30-32]. However none of the studies, thus far, have demonstrated whether long-term astrocytic reactivity can facilitate recovery of synaptic activity after loss of afferent activity. Further studies are needed to pinpoint the role of glial activation after loss of sensory afferents, and to determine, for example, whether astrocytes have a role in initiating neurodegeneration. Interestingly, Pekny et al. [60] suggest that reactive astrocytes may play an important role in neuroprotection in the early stages of injury, but may also inhibit CNS plasticity at a later stage after injury.

In addition to astrocyte activation, we observed a microglial response within 28 days after ONT. The microglial activation was robust in the ONT, but not OHT, brains, and predictable given that nerve transections are known to cause anterograde neurodegeneration. While we did not observe a microglial response in the CNS after OHT in the primate model, Wang et al. reported microglial activation in rodents with elevated IOP [49]. Differences in species as well as the method of inducing elevated IOP used in Wang et al.’s study may account for the observed differences in the microglial activation in the rodent LGN [49]. Microglial activation has also been reported in the optic nerve head of experimental glaucoma and implicated in the loss of retinal ganglion cells in both animal models [61-63] and in human glaucoma [64,65]. Because of the significant microglial response in the retina after elevated IOP, it was suggested that targeting microglial activation may be effective at rescuing retinal ganglion cells from atrophy [66-68]. Interestingly, however, in our study there was no measurable microglial response in the LGN or V1 of the OHT brains even after durations as long as 58 days post elevated IOP. As microglia respond principally to injury or neuronal damage and remove neuronal debris, the lack of their activation in our short elevated IOP animals suggest cell loss has not occurred yet. Other studies of longer elevated IOP duration have shown neuronal cell loss [16,18-22,27,28,46]. The absence of neuronal loss, and other irreversible events, at two months suggests that it may be desirable to develop treatments that can be used soon after the onset of glaucoma, which will protect against late stage neurodegenerative cell loss. It will be important to assess the microglial and astrocyte response in the LGN and V1 after unilateral OHT lasting beyond the two months duration studied here, to identify the sequence of cellular events that occur after astrocyte activation and before neuronal cell loss. Sasaoka et al. [69] reported GFAP immunoreactivity as well as neuronal cell loss in the LGN after 11–18 weeks of unilateral OHT in two cynomolgus monkeys. While Sasaoka et al. [69] did not also assess for microglial activation in this study, they did assess microglia activation in a subsequent study and concluded that microglial activation is detectable in positron emission tomography after unilateral OHT in a primate model [70]. Thus, assessing metabolic activity, astrocyte reactivity, or microglial activation by noninvasive imaging of the CNS may be helpful in the early detection of central changes associated with glaucoma that cannot be assessed at the level of the retina.
